# Inhalable particulate matter and mitochondrial DNA copy number in highly exposed individuals in Beijing, China: a repeated-measure study

**DOI:** 10.1186/1743-8977-10-17

**Published:** 2013-04-29

**Authors:** Lifang Hou, Xiao Zhang, Laura Dioni, Francesco Barretta, Chang Dou, Yinan Zheng, Mirjam Hoxha, Pier Alberto Bertazzi, Joel Schwartz, Shanshan Wu, Sheng Wang, Andrea A Baccarelli

**Affiliations:** 1 Department of Preventive Medicine Feinberg, School of Medicine Northwestern University, 680 North Lake Shore Drive, Suite 1400, Chicago, IL 60611, USA; 2 Robert H. Lurie Comprehensive Cancer Center Feinberg School of Medicine, Northwestern University, Chicago, IL, USA; 3 Department of Clinical Sciences and Community Health, University of Milan, Milan, Italy; 4 Epidemiology Unit, Department of Preventive Medicine, Foundation IRCCS Cà Granda Ospedale Maggiore Policlinico, Milan, Italy; 5 Department of Safety Engineering, China Institute of Industrial Relations, Beijing, China; 6 Department of Environmental Health, Harvard School of Public Health, Boston, MA, USA; 7 Department of Occupational Health, SINOPEC Research Institute of Safety Engineering, Qingdao, China; 8 Department of Occupational and Environmental, Health Peking University Health Science, Center No. 38 Xueyuan Road Haidian District, Beijing 100191, China

**Keywords:** China, Mitochondrial DNA, Mitochondrial DNA copy number, Particulate matter, Traffic pollution

## Abstract

**Background:**

Mitochondria are both a sensitive target and a primary source of oxidative stress, a key pathway of air particulate matter (PM)-associated diseases. Mitochondrial DNA copy number (MtDNAcn) is a marker of mitochondrial damage and malfunctioning. We evaluated whether ambient PM exposure affects MtDNAcn in a highly-exposed population in Beijing, China.

**Methods:**

The Beijing Truck Driver Air Pollution Study was conducted shortly before the 2008 Beijing Olympic Games (June 15-July 27, 2008) and included 60 truck drivers and 60 office workers. Personal PM_2.5_ and elemental carbon (EC, a tracer of traffic particles) were measured during work hours using portable monitors. Post-work blood samples were obtained on two different days. Ambient PM_10_ was averaged from 27 monitoring stations in Beijing. Blood MtDNAcn was determined by real-time PCR and examined in association with particle levels using mixed-effect models.

**Results:**

In all participants combined, MtDNAcn was negatively associated with personal EC level measured during work hours (β=−0.059, 95% CI: -0.011; -0.0006, p=0.03); and 5-day (β=−0.017, 95% CI: -0.029;-0.005, p=0.01) and 8-day average ambient PM_10_ (β=−0.008, 95% CI: -0.043; -0.008, p=0.004) after adjusting for possible confounding factors, including study groups. MtDNAcn was also negatively associated among office workers with EC (β=−0.012, 95% CI: -0.022;-0.002, p=0.02) and 8-day average ambient PM_10_ (β=−0.030, 95% CI: -0.051;-0.008, p=0.007).

**Conclusions:**

We observed decreased blood MtDNAcn in association with increased exposure to EC during work hours and recent ambient PM_10_ exposure. Our results suggest that MtDNAcn may be influenced by particle exposures. Further studies are required to determine the roles of MtDNAcn in the etiology of particle-related diseases.

## Background

Ambient exposure to particulate matter (PM) has been linked with increased hospitalization and mortality from respiratory and cardiovascular diseases [1]. Short exposures to high levels of PM have been suggested to trigger acute events, such as acute myocardial infarction and asthma attacks [2,3]. Longer and protracted PM exposures have been shown to increase the risk of chronic diseases such as atherosclerosis, chronic obstructive pulmonary disease, and lung cancer [2,4-6]. In vitro and in vivo studies have found the generation of reactive oxygen species (ROS) and increased oxidative stress as a primary biological process for air pollution-related adverse health effects [7,8].

Mitochondria are the major intracellular source and primary target of ROS. Each human cell contains between several hundred and over a thousand mitochondria, each carrying 2–10 copies of mitochondrial DNA (MtDNA). MtDNA copy number (MtDNAcn) is correlated with the size and number of mitochondria, which have been shown to change under different energy demands, as well as different physiological or environmental conditions [9]. Decreased blood MtDNA content has been associated with various diseases or conditions, such as diabetes and insulin resistance [10-13], glucose dysregulation [14], cognitive function [15], osteoporosis [16-18], renal cell carcinoma [19], and hypertension [12,20]. Evidence on environmental exposure and MtDNAcn has begun to accumulate, showing increased blood MtDNAcn in relation to exposure to benzene [21,22] and ambient PM [23], decreased blood MtDNAcn in relation to smoking [24], and decreased placenta MtDNAcn in relation to prenatal exposure to PM_10_[25] and maternal smoking [26]. These findings suggest that mitochondrial damage and dysfunction, as reflected in MtDNAcn, may represent a biological effect along the path linking PM inhalation to its health effects.

Beijing, China, has some of the highest levels of air pollution in the world [27]. Traffic-derived air pollution is critical in Beijing due to its very high population density, rapid increase in vehicular traffic, and limited emission control. Investigating individuals exposed to high levels of PM, such as those in Beijing, may help identify MtDNAcn changes that cannot be easily identified in populations with lower exposures. In the present study, we measured blood MtDNAcn in truck drivers and indoor office workers in Beijing to investigate the association of blood MtDNAcn with exposure to PM.

## Material and methods

### Study population and design

The Beijing Truck Driver Air Pollution Study (BTDAS), conducted between June 15 and July 27, 2008, included 60 truck drivers and 60 indoor office workers. All study participants worked and lived in the Beijing metropolitan area and had been on their current jobs for at least two years. No subject was on regular medication, including anti-inflammatory drugs or aspirin during the course of the study. Because PM levels are highly variable on a day-to-day basis, we examined all participants on two work days separated by 1–2 week periods. Truck drivers and indoor office workers were matched by sex, smoking, and education, and partially matched (5-year intervals) by age. In-person interviews using a detailed questionnaire were conducted to collect information on demographics, lifestyle, and other exposures. Information on time-varying factors, including tea, alcohol, and smoking, was obtained for past usual exposure, as well as on each examination day. Individual written informed consent was obtained from all participants prior to enrollment in the study. Institutional Review Board approval at all participating institutions was obtained prior to study participant recruitment.

### Personal PM_2.5_ exposure measurements

We measured average personal PM_2.5_ on both examination days using gravimetric samplers worn by the study participants during the 8 hours of work. The air sampler was carried in a belt pack with the inlet clipped near the breathing zone. Each air sampler setup included an Apex pump (Casella Inc., Bedford, UK), a Triplex Sharp-Cut Cyclone (BGI Inc., Waltham, Massachusetts, USA), and a 37-mm Teflon filter placed on top of a drain disc and inside a metal filter holder. The filters were kept under atmosphere-controlled conditions before and after sampling and were weighed with a microbalance (Mettler-Toledo Inc., Columbus, Ohio, USA). A time-weighted average of PM_2.5_ concentration was recorded by dividing the change in filter weight before and after the sampling by the volume of air sampled. We found high reproducibility of PM_2.5_ measures (r=0.944) in replicate measures on a subset of 24 participants who wore two monitors at the same time (see Additional file 1: Figure S1). The blackness of the same filters used to measure PM_2.5_ was assessed using an EEL Model M43D Smokestain Reflectometer, applying the standard black-smoke index calculations of the absorption coefficients based on reflectance [28]. We assumed a factor of 1.0 for converting the absorption coefficient to elemental carbon (EC) mass [29,30], which was then divided by the sampled air volume to calculate average EC exposure concentration [28]. EC is a combustion by-product contained in PM that has been used as a surrogate measure for PM from gasoline- and especially diesel-powered motor vehicles [29].

### Ambient PM_10_ data

Ambient PM_10_ data during the study period were obtained from the Beijing Municipal Environmental Bureau (http://www.bjepb.gov.cn/air2008/Air.aspx). We used daily averages of PM_10_ levels computed from data obtained from 27 monitoring stations to estimate the average PM_10_ level in Beijing. The locations of the 27 monitoring stations were distributed across the Beijing city area to generate an average representative of the entire city. We used the ambient PM_10_ data as an indicator of exposure in the days immediately before the examination days, and used multiple averaging time windows, which included 1-day mean (24-hour average of the examination day), 2-day mean (average of the examination day and 1 day before the examination), 5-day mean (average of the examination day and 4 days before the examination), and 8-day mean (average of the examination day and 7 days before the examination).

### Analysis of Relative MtDNAcn

All samples were processed, collected, and analyzed using standardized protocols. We used EDTA tubes to collect 7 ml of whole blood that was promptly centrifuged at 2500 rpm for 15 minutes. Total DNA was extracted from 200 μl buffy coat, using the Wizard Genomic DNA purification kit (Promega, Madison, WI). MtDNAcn was measured by a quantitative real-time polymerase chain reaction (qPCR) assay, as previously described [19,31]. The assay measures MtDNAcn in experimental samples compared to a standard DNA sample used as reference in all the reactions. A relative value equal to 1 indicates that the experimental sample analyzed has MtDNAcn equal to that of the standard reference DNA sample used in the analysis. The assay is based on determinations of the ratio of mitochondrial (Mt) copy number to single copy gene (human [beta] globin: hbg) copy number, which is measured on both the experimental samples and the standard reference DNA. The Mt PCR mix was: iTaq™ SYBR® Green Supermix with ROX (Bio-Rad) 1x, MtF3212 500 nM (primer), MtR3319 500 nM, EDTA 1x. The hbg PCR mix was: iTaq SYBR Green Supermix (Bio-Rad) 1x, hbgF 500 nM (primer), hbgR 500 nM, EDTA 1x. 4 ng DNA was loaded in a 10 μl PCR reaction. We used MtDNA and hbg primers as previously described [23,32]. For the reference DNA, we used pooled DNA from 20 participants randomly selected from our study participants (500 ng for each sample) to generate in every Mt and S PCR run a fresh standard curve, which ranged from 20 ng/μl to 0.25 ng/μl. The mean slopes of standard curve for Mt and S were −3.51 and −3.42 respectively, and the R^2^ for each standard curve was 0.99 or greater. Standard deviations for the cycle of threshold (Ct) value were accepted at 0.25. Otherwise, the test was repeated. All PCRs were performed on the 7900 Fast Real-Time PCR System (Applied Biosystems, Foster City, CA). The thermal cycling conditions for the MtDNA PCR were 2 minutes at 50°C and 3 minutes at 95°C to activate the hot-start iTaq DNA polymerase, followed by 35 cycles comprised of 15 s denaturation at 95°C and 60 s anneal/extend at 60°C. The thermal cycling conditions for the hbg PCR were 3 minutes at 95°C to activate the hot-start iTaq DNA polymerase, followed by 35 cycles comprised of 15s denaturation at 95°C and 60s anneal/extend at 58°C. Each run was completed by melting curve analysis to confirm the amplification specificity and absence of primer dimers. All the laboratory personnel performing the experiments described above were blinded to the exposure status of the DNA samples.

All samples were randomized in each PCR plate, and the samples obtained on two different days of each subject were kept on the same plate. All samples were run in triplicates in 384-well plates. The average of the three Mt measurements was divided by the average of the three S measurements to calculate the Mt/S ratio for each sample. The coefficient of variation for the Mt/S ratio in duplicate samples analyzed on two different days was 8%.

### Statistical analysis

Standard descriptive statistics were used to present the characteristics of truck drivers and office workers. Differences in participant characteristics between the two groups were tested using Student’s t-tests and Fisher’s exact tests. Because MtDNAcn was measured twice for each participant on two work days separated by 1–2 week periods, we used mixed-effects models to regress MtDNAcn over the occupational group (0, office workers; 1, truck drivers) and test for differences between groups and estimate group-specific means and standard deviations (SDs). We fitted unadjusted models, as well as models adjusted for variables either matched or not completely matched between the two groups, i.e. sex (male, female), smoking (never, defined as those who have never smoked a cigarette or who smoked fewer than 100 cigarettes in their entire lifetime; former, defined as those who have smoked at least 100 cigarettes in their lifetime, but currently do not smoke; and current, defined as those who were currently smoking), age (continuous), body mass index (BMI) (continuous), work hours per week (continuous), and pack-year of cigarette smoking (continuous). For analyses on all participants, we further adjusted for study groups (truck drivers vs. office workers). The mixed-effect regression models were:

$$Y=\beta_{0}+\beta_{1}\left(\mathrm{Group}\right)+\beta_{2}X_{2}+...+\beta_{n}X_{n}+\xi +e$$

where β_0_ is the overall intercept; β_1_ is the regression coefficient for the group; β_2_… β_n_ are the regression coefficients for the covariates included in multivariate models; ξ is the random effect for the participant, and *e* is the residual error term.

We also evaluated the associations of personal PM_2.5_, EC, and ambient PM_10_ (1-day, 2-day, 5-day, or 8-day means) with MtDNAcn using mixed-effects models adjusted for group, age, sex, BMI, smoking status, pack-years of cigarette smoking, and work hours per week. To optimize power, we conducted primary analyses on the association of exposure measures and MtDNAcn by fitting these models to all participants combined. Secondarily, we evaluated associations in office workers or truck drivers separately. The mixed-effects models were:

$$Y=\beta_{0}+\beta_{1}\left(\mathrm{Exp}\right)+\beta_{2}X_{2}+...+\beta_{n}X_{n}+\xi +e$$

where β_0_ is the overall intercept; β_1_ is the regression coefficient for the exposure variable (PM_2.5,_ EC, or PM_10_); β_2_ . . . β_n_ are the regression coefficients for the covariates included in multivariate models; ξ is the random effect for the participant, and *e* is the residual error term. All tests were 2-sided and an alpha level <0.05 was considered significant. All analyses were performed in SAS 9.2 (SAS Institute Inc., Cary, NC).

## Results

Table 1 shows the characteristics of the 60 office workers and 60 truck drivers. Truck drivers were moderately but significantly older than office workers. Truck drivers had higher BMI and reported a higher number of pack-years of smoking and longer work hours per week during the study period.

**Table 1:** Characteristics of the study participants

	**Office workers**	**Truck drivers**	**p-value**^**a**^
	**(n=60)**	**(n=60)**	
**Age [years], mean ± SD**	30.27 ± 7.96	33.53 ± 5.65	0.01
**Sex, n (%)**			
Male	40 (66.67)	40 (66.67)	
Female	20 (33.33)	20 (33.33)	1.00
**BMI [kg/m**^**2**^**], mean ± SD**	22.76 ± 3.38	24.27 ± 3.21	0.01
**Smoking, n (%)**			
Never smoker	35 (58.33)	34 (56.67)	
Former	2 (3.33)	2 (3.33)	
Current	23 (38.33)	24 (40)	1.00
**Pack-years of smoking, mean ± SD**^**b**^	1.2 ± 2.7	5.1 ± 9.3	0.003
**Work hours per week, mean ± SD**	50.6 ± 11.0	67.3 ± 14.0	<0.001
**Day of the week on the two study days, n (%)**			
Monday	16 (13.33)	19 (15.83)	
Tuesday	18 (15)	13 (10.83)	
Wednesday	14 (11.67)	15 (12.5)	
Thursday	15 (12.5)	20 (16.67)	
Friday	17 (14.17)	19 (15.83)	
Saturday	18 (15)	16 (13.33)	
Sunday	22 (18.33)	18 (15)	0.88^**c**^
**Average temperature on the two study days, mean ± SD**	25.36 ± 2.51	25.34 ± 2.51	0.96^**c**^
**Average dew point on the two study days, mean ± SD**	20.63 ± 2.06	20.61 ± 2.13	0.93^**c**^

^a^ P-values were calculated using Student’s t-test and Fisher’s exact test for continuous and categorical variables, respectively.

^b^ Only current or former smokers.

^c^ Cumulative over the two study days. Based on 240 total observations (120 study days for office workers and 120 study days for truck drivers). P-values were obtained from mixed-effects regression models.

As shown in Table 2, average personal PM_2.5_ was 126.8 μg/m^3^ (SD=68.8 μg/m^3^) in truck drivers and 94.6 μg/m^3^ (SD=64.9 μg/m^3^) for office workers (p<0.001). Average personal EC was 17.2 μg/m^3^ (SD=6.6 μg/m^3^) in truck drivers and 13.0 μg/m^3^ (SD=4.0 μg/m^3^) for office workers (p<0.001). As expected, the levels of ambient PM_10_ in the city of Beijing on the days before the examinations (1–8 day means) did not differ between truck drivers and office workers.

**Table 2:** **Levels of personal exposure to PM**_**2.5 **_**and Elemental Carbon (EC) during work hours, and of ambient PM**_**10 **_**on and before the examination days**

**Time window**	**Office workers**								**Truck drivers**									
	**N**	**Mean**	**SD**	**10pct**	**25pct**	**Median**	**75pct**	**90pct**	**N**	**Mean**	**SD**	**10pct**	**25pct**	**Median**	**75pct**	**90pct**	**p-value**	
**Personal PM**_**2.5**_**(μg/m**^**3**^**) on the examination days, from personal monitors**^**a**^																		
8 hours	120	94.6	64.9	22.4	48.5	86.2	126.6	183.4	119	126.8	68.8	46.3	73.9	116.8	160.5	213.9	<0.001	
**Personal EC (μg/m**^**3**^**) on the examination days, from personal monitors**^**a**^																		
8 hours	118	13.0	4.0	7.1	10.0	13.2	15.8	18.4	120	17.2	6.6	9.2	12.9	16.7	20.9	26.1	<0.001	
**Ambient PM**_**10**_**(μg/m**^**3**^**) from ambient monitors on the days prior to the examination days**																		
1-day mean	120	121.5	47.8	72.0	82.0	118.0	146.0	186.0	120	119.5	51.2	64.0	82.0	118.0	142.0	188.0	0.76	
2-day mean	120	121.6	38.0	74.5	93.0	125.0	146.0	173.0	120	119.3	40.3	66.0	91.0	120.0	144.0	157.0	0.64	
5-day mean	120	119.5	26.9	80.7	105.6	119.6	138.0	148.8	120	118.2	25.6	81.0	96.8	119.6	136.8	144.0	0.69	
8-day mean	120	119.5	23.0	84.9	101.8	119.9	141.5	146.5	120	120.2	21.5	95.6	102.8	120.4	139.0	146.3		0.81

^a^ Measured during the work hours of examination days using lightweight personal monitors.

In unadjusted analyses, mean MtDNAcn was 1.04 (SD=0.22) in truck drivers and 1.05 (SD=0.22) in office workers (p=0.73). In multiple regression models adjusted for sex, age, BMI, smoking status, work-hour week, and pack-years of smoking, the estimated mean MtDNAcn did not differ between truck drivers (1.05, SD=0.24) and office workers (1.09, SD=0.25) (p=0.40) (Table 3). Our further analysis on measurement of MtDNAcn on each work day by study groups resulted in comparable results (data not shown). We also compared the MtDNAcn between males and females, finding that the mean MtDNAcn is significantly higher in females (1.14, SD=0.59) than in males (0.98, SD=0.25) (p=0.002) in multiple regression model (see Additional file 1: Table S1).

**Table 3:** **Mean mitochondrial DNA copy number** (**MtDNAcn**) **in truck drivers and office workers**

	**Office workers(obs**=**120)**	**Truck drivers(obs**=**120)**	**p**-**value**
	**Mean** ± **SD**	**Mean** ± **SD**	
**Unadjusted**	1.05 ± 0.22	1.04 ± 0.22	0.73
**Adjusted**^**a**^	1.09 ± 0.25	1.05 ± 0.24	0.40

^a^ Adjusted for age, sex, BMI, pack-years, smoking status (never, former, current), and work hours per week.

Table 4 shows the associations of MtDNAcn with the levels of personal PM_2.5_, EC, and ambient PM_10_. Personal PM_2.5_ measured during work hours did not show any significant association with MtDNAcn in all participants combined, office workers, or truck drivers. In all participants combined, MtDNAcn decreased in association with higher EC measured during work hours (β=−0.059, 95% CI: -0.011;-0.0006, p=0.03). In office workers, MtDNAcn was also negatively associated with EC measured during work hours (β=−0.012, 95% CI: -0.022;-0.002, p=0.02), whereas in truck drivers the negative effect was smaller and not significant. In all participants combined, MtDNAcn decreased in association with increased levels of 5-day and 8-day means of ambient PM_10_. Each 10 μg/m^3^ increase in the 5-day mean of ambient PM_10_ was associated with an average decrease of 0.017 relative units in MtDNAcn (95% CI: -0.029;-0.005, p=0.01) in the analyses on all study participants. Each 10 μg/m^3^ increase in the 8-day mean of ambient PM_10_ was associated with an average decrease of 0.008 relative units in MtDNAcn (95% CI: -0.043;-0.008, p=0.004) in the analyses on all study participants. Stratified analyses in office workers and truck drivers showed the associations of the 5- and 8-day means of ambient PM_10_ with MtDNAcn in each of the two groups, although it was statistically significant only in office workers for the 8-day mean of ambient PM_10_ (β=−0.030, 95% CI: -0.051;-0.008, p=0.007) (Table 4). Our stratified analysis by sex showed that the effect of exposure, including personal EC and PM_10_ (2-day, 5-day, and 8-day means), on MtDNAcn was stronger in men than in women (see Additional file 1: Table S2).

**Table 4:** **Effect of ambient particles on mitochondrial DNA copy number**^**a **^**at the end of the work shift**, **by group and on all subjects**

		**All subjects(obs**=**240)**^**b**,**c**^			**Office workers(obs**=**120)**			**Truck drivers(obs**=**120)**^**d**^		
		**β**	**(95%CI)**	**p**-**value**	**β**	**(95%CI)**	**p**-**value**	**β**	**(95%CI)**	**p**-**value**
**Mitochondrial DNA Copy Number (%)**										
	Personal PM_2.5_ (work hours)	0.001	(−0.003;0.006)	0.55	0.001	(−0.006;0.007)	0.81	0.005	(−0.002;0.012)	0.18
	Personal EC (work hours)	−0.059	(−0.011;-0.006)	0.03	−0.012	(−0.022;-0.002)	0.02	−0.004	(−0.010;0.003)	0.28
	Ambient PM_10_ (1-day mean)	−0.005	(−0.011;0.002)	0.18	−0.004	(−0.012;0.004)	0.28	−0.003	(−0.011;0.004)	0.40
	Ambient PM_10_ (2-day mean)	−0.006	(−0.014;0.002)	0.12	−0.006	(−0.012;0.003)	0.19	−0.002	(−0.012;0.008)	0.67
	Ambient PM_10_ (5-day mean)	−0.017	(−0.029;-0.005)	0.01	−0.011	(−0.025;0.004)	0.15	−0.012	(−0.028;0.004)	0.14
	Ambient PM_10_ (8-day mean)	−0.008	(−0.043;-0.008)	0.004	−0.030	(−0.051;-0.008)	0.007	−0.012	(−0.036;0.013)	0.35

^a^ Adjusted for age, sex, BMI, smoking status (never, former, current), pack-years of smoking, and work hours per week.

^b^ Adjusted for group, age, sex, BMI, smoking status (never, former, current), pack-years of smoking, and work hours per week.

^c^ For PM_2.5_ exposure, statistics are estimated on 239 observations because of a missing value in truck drivers group in work day 2.

^d^ For PM_2.5_ exposure, statistics are estimated on 119 observations because of a missing value in work day 2.

We previously examined the effects of PM exposure on blood pressure (BP) in this study population, finding a delayed effect of ambient PM_10_ on BP [33]. In this study, we examined the correlations of MtDNAcn with BP and other risk factors. We observed an inverse association of MtDNAcn with systolic (p=0.002) and diastolic BP (p<0.001), as well as with BMI (p=0.049) and pack-years of smoking (p=0.038) (see Additional file 1: Table S3).

## Discussion

The present study showed lower MtDNAcn in association with increased levels of personal EC measured during work hours and of ambient PM_10_ averaged over 5 and 8 days before the MtDNAcn examination days. We found no significant associations of MtDNAcn with personal measures of PM_2.5_ taken during work hours on the day of the examination, nor with PM_10_ on the examination day and the 2-day mean. MtDNAcn was similar in truck drivers and office workers. Thus our results do not support effects of work-related long-term differences in exposure to air particles.

Research on MtDNAcn in relation to air pollution is still limited with inconsistent results. Two studies have demonstrated that individuals exposed to higher ambient benzene exhibited higher MtDNAcn than participants with lower exposure [21,22]. In our previous study of healthy steel workers in Northern Italy, personal PM_10_ and PM_1_ were associated with increased MtDNAcn [23]. In this group of Italian steel foundry workers with high exposure to metal-rich PM, MtDNAcn was determined from blood DNA obtained on the 1^st^ and 4^th^ day of the same work week after two off-work days [23]. Exposures to PM_10_, PM_1_, and coarse particles showed a dose–response relationship with increased MtDNAcn on both the 1^st^ and 4^th^ days of the study week, indicating that the correlations between PM exposure and MtDNAcn were the result of a more protracted exposure to PM, rather than of the acute exposure between the two days. In the present study, we observed significant negative association of 5- and 8-day means of ambient PM_10_ with MtDNAcn in office workers, but not in truck drivers. It should be noted that the dose–response slope between particles and cardiovascular mortality has been shown to be nonlinear, with lower slopes at higher particle concentrations [34]. Therefore, PM effects might be substantial at low- to middle-range doses and taper off at higher concentrations. Also, these results suggest that MtDNA may react differently under the influence of different environmental factors, leading to differences in copy numbers. Compared with the present study, higher PM_10_ level (median=179.44 μg/m^3^), metal components (i.e., chromium, lead, arsenic, nickel, manganese), older age (mean=44 years), and different race in steel foundry workers might have all contributed to the discrepancy in the results between the two studies. In the present study, levels of EC during the work hours, taken as a tracer of exposure to traffic particles, were associated with decreased MtDNAcn. This finding indicated effects on MtDNAcn from traffic particles that appeared on the same day of the study. The finding of stronger effects of EC in office workers than that in truck drivers suggests that the decrease in MtDNAcn did not result from the effects of work-related exposure to traffic air particles. Compared to truck drivers, the office workers may represent a more homogenous group in relation to different variables, such as socioeconomic status, smoking, and other factors that may have the potential to influence MtDNAcn. Also, unmeasured exposures or differences in particle content may have influenced MtDNAcn more in office workers than in truck drivers. However, we conducted a test for interaction to determine whether the effects of air particle exposures on MtDNAcn in office workers were different from those in truck drivers. We did not observe any significant interaction (p>0.05) (data not shown), indicating no significant difference in the slope of the relation between air particle exposures and MtDNAcn between office workers and truck drivers. Therefore, the observed stronger association and larger effect in office workers than truck drivers may mostly reflect imprecision in the regression estimates due to limited sample size. Larger studies are warranted in larger groups with different sources of exposure.

MtDNAcn is dependent on oxidative stress level, cell antioxidant capacity, and quality of mitochondria and MtDNA [35]. Mild oxidative stress may stimulate MtDNAcn synthesis, while high exposure may result in decreased or no synthesis, owing to severe oxidative damage of cells. PM exposure, particularly from traffic sources, induces systemic inflammation [36], which may lead to decreased MtDNAcn. Haden et al. found that MtDNAcn was reduced in the early phase of sepsis as a consequence of the sepsis-induced oxidative damage and inflammation in mice [37]. Pyle et al. observed decreased MtDNAcn in the blood of sepsis patients compared with controls [38].

MtDNA is dynamic and can be influenced by multiple factors. We observed inverse associations of MtDNAcn with factors that can cause oxidative stress, including cigarette smoking and BMI (see Additional file 1: Table S3). The observed negative association of MtDNAcn with pack-years of smoking is consistent with previous findings that have associated pack-years of smoking with lower blood MtDNAcn in men [24]. We also found a negative association of BMI with MtDNAcn. A strong inverse association was reported between BMI and MtDNAcn in adipocytes in healthy subjects [39]. In this study population, we have previously reported an association of increased BP with ambient PM_10_ over 5 or 8 days before the examination days [33]. In the present analysis, we observed decreased MtDNAcn with increasing systolic and diastolic BP (see Additional file 1: Table S3). This finding is in line with previous studies showing a negative correlation between MtDNAcn in blood and BP [12,20]. Also, previous investigations have associated decreased blood MtDNAcn with PM-related diseases, such as cognitive function [15]. Decreased MtDNAcn in placental tissues has been associated with prenatal PM_10_ exposure (mean=22.7 ± 3.7 in whole pregnancy) [25] and maternal smoking [26].

Our additional analysis on male and female have found higher mtDNAcn in women than that in men, which is consistent with two previous studies [40,41]. Thyagarajan et al. found that individuals with high dietary intake of α- and β-carotenes had slightly higher MtDNAcn, and suggested that lifestyle factors may contribute to the gender-related difference [40]. However, the exact mechanisms underlying the sex-related differences are not well understood. Further, in our stratified analysis on the effect of exposures by sex, we found that the effect of exposure, including personal EC and PM_10_ (2-day, 5-day, and 8-day means), on MtDNAcn appears to be stronger in men than in women. Similary, Purdue et al. has reported a stronger association of MtDNAcn with renal cell carcinoma in men than women [41]. However, due to the limited sample size, particularly, in the female group, our results need to be interpreted with caution. Larger studies are needed to examine sex-differences in susceptibility toward air pollution in the future.

Some previous investigations have suggested that MtDNA has a long-half life and low turnover rates. For instance, Collins et al. showed a half-life of approximately 350 days for MtDNA in rats [42]. However, other studies have shown more rapid half-lives, varying between 7 and 31 days [43]. It has been postulated that the MtDNA turnover rates are variable depending on the tissue investigated as well as the effects of environmental factors [44]. Our data indicate that relatively rapid changes in MtDNAcn may occur in a cell type with high turnover rates, such as neutrophils with an average half-life of 5–6 days [45], at high levels of exposure to air particles.

Our study had the advantage of having both personal and ambient measures of air pollution. Our technical validation of personal PM_2.5_ measures showed high reproducibility (r=0.944). We also recognize that our study is subject to a number of limitations. In our investigation, we did not examine inflammatory markers, which may play important roles in both mtDNAcn changes and PM-related diseases, thus limiting our ability of understanding how inflammation may affect PM-related MtDNAcn changes. Further studies are needed to examine cytokines, in particular, IL2, IL4, IL6, IL8, and TNFα that have been previously associated with PM exposure [46-48], and high-sensitivity C-reactive protein (hs-CRP) [49] to clarify the role of inflammation in the relation of MtDNAcn with PM exposure. Because of the relatively small sample size, we cannot exclude false negative or chance findings. In addition to using personal PM_2.5_ measures, we used stationary measures of ambient PM_10_ to represent exposures, which are just a proxy of personal exposure. However, simulation studies have shown that the error introduced by using data from stationary monitors is highly unlikely to bias away from the null, and indicated that this exposure misclassification may lead to an underestimation of the health effects of air pollution [50]. In addition, serial measures of ambient particulate concentrations have been shown to be representative of variations in personal exposures [51], particularly in the presence of high ambient PM levels [52]. The observed decreased MtDNAcn in our study suggests that high doses of exposures, such as those found in Beijing, might lead to clearance of cells with highly damaged mitochondria. However, the clinical significance of such small changes in MtDNA content remains largely unknown. More studies are warrented to examine the risk of future disease in large cohorts with clinical disease outcomes.

Overall, our investigation provided evidence that short-term exposure to air pollution is associated with decreased MtDNAcn. These findings suggest that damaged mitochondrial DNA, as reflected in peripheral blood MtDNAcn, may reflect recent toxic exposures and potentially contribute to the etiology of PM-related diseases. Further studies are required to validate the present findings, as well as to better elucidate the time relationships between PM exposure and MtDNAcn.

## Abbreviations

BMI: Body mass index; BP: Blood pressure; BTDAS: Beijing Truck Driver Air Pollution Study; CI: Confidence interval; EC: Elemental carbon; MtDNA: Mitochondrial DNA; MtDNAcn: MtDNA copy number; qPCR: Quantitative real-time polymerase chain reaction; PM: Particulate matter; PM2.5: Particulate matter ≤2.5 μm; PM10: Particulate matter ≤10 μm; ROS: Reactive oxygen species; SD: Standard deviation

## Competing interests

The authors declare that they have no competing interests.

## Authors’ contributions

Hou, Schwartz, Wang, and Baccarelli generated the study concept, conducted studies and supervised laboratory assays, data analysis and interpretation, participated in manuscript writing. Hou and Zhang contributed to data interpretation and manuscript writing. Hoxha and Dioni performed the laboratory analyses. Wang, Dou, and Wu were involved in study conduction. Barretta, Zheng, and Bertazzi performed the statistical analysis. All authors read and approved the final manuscript.

## Supplementary Material

Additional file 1: Figure S1 Measures of PM_2.5_ from two independent personal monitors worn at the same time by a subset of 12 study subjects to test the accuracy of the measurements. **Table S1.** Pearson’s Correlation Coefficient between MtDNAcn and risk factors. **Table S2.** Pearson’s correlation coefficient among mtDNAcn, age, smoking, BMI and blood pressure. **Table S3.** Correlation between MtDNAcn and risk factors in bivariate regressions model. **Table S4.** Correlation between MtDNAcn and risk factors in multiple regression model. Click here for file
