# Supplementary material for: Inhalable particulate matter and mitochondrial DNA copy number in highly exposed individuals in Beijing, China: a repeated-measure study
Source: Part Fibre Toxicol. 2013 Apr 29;10:17. doi: 10.1186/1743-8977-10-17 (PMC3649952; doi:10.1186/1743-8977-10-17)
Supplement: Additional file 1: Figure S1 — Measures of PM2.5 from two independent personal monitors worn at the same time by a subset of 12 study subjects to test the accuracy of the measurements. Table S1. Pearson’s Correlation Coefficient between MtDNAcn and risk factors. Table S2. Pearson’s correlation coefficient among mtDNAcn, age, smoking, BMI and blood pressure. Table S3. Correlation between MtDNAcn and risk factors in bivariate regressions model. Table S4. Correlation between MtDNAcn and risk factors in multiple regression model. [file 1743-8977-10-17-S1.doc]

**Additional Files**

Additional File 1

**
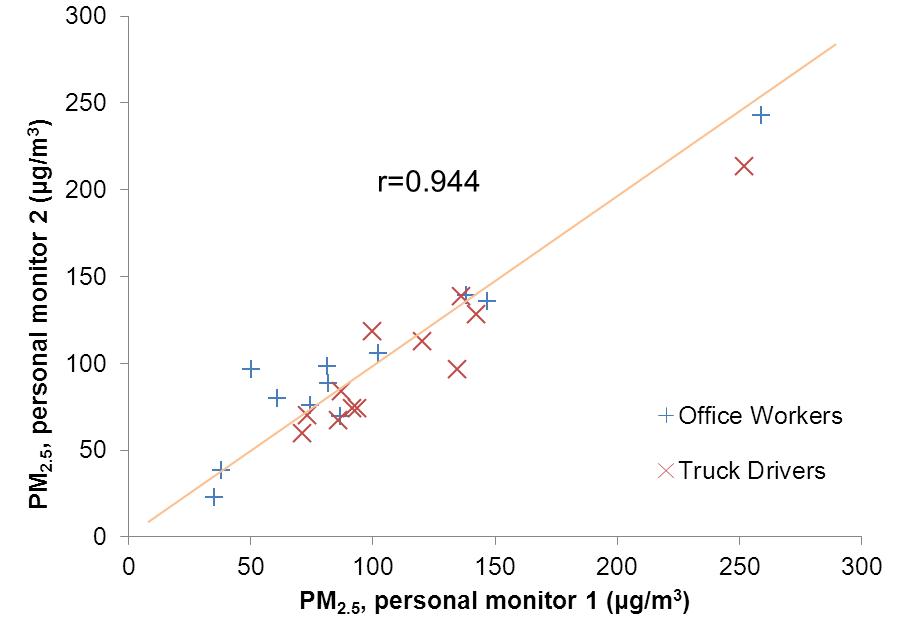
**

**Figure S1.** Measures of PM_2.5_ from two independent personal monitors worn at the same time by a subset of 12 study subjects to test the accuracy of the measurements. The scatter plot shows the high correlation (r=0.944) between monitor 1 and monitor 2.

**Additional File 2**

**Table S1. Mean mitochondrial DNA copy number (MtDNAcn) in males and females**

|  | **Males**  **(obs=120)** |  | **Females**  **(obs=120)** | **p-value** |
| --- | --- | --- | --- | --- |
|  | **Mean±SD** |  | **Mean±SD** |  |
| **Unadjusted** | 0.98±0.25 |  | 1.16±0.35 | <0.001 |
| **Adjusted^a^** | 0.99±0.43 |  | 1.14±0.59 | 0.002 |

^a^ Adjusted for age, BMI, pack-year, smoking status during study time, and work hour week.

Table S2. Stratified analysis for sex of effect of ambient on MtDNAcn

|  | **All Subjects**  **(obs=80)** | | |  | **Office Workers**  **(obs=40)** | | |  | **Truck Drivers**  **(obs=40)** | | |
| --- | --- | --- | --- | --- | --- | --- | --- | --- | --- | --- | --- |
|
|  | **β** | **(95% CI)** | **p** |  | **β** | **(95% CI)** | **p** |  | **β** | **(95% CI)** | **p** |
| **Mitochondrial DNA Copy Number (%) in males** | | | | | | | | | | | |
| Personal PM_2.5_ (work hours) | 0.002 | (-0.003;-0.007) | 0.397 |  | 0.002 | (-0.004;0.007) | 0.604 |  | 0.004 | (-0.004;0.011) | 0.303 |
| Personal EC (work hours) | -0.061 | (-0.120;-0.002) | **0.047** |  | -0.119 | (-0.234;0.004) | **0.077** |  | -0.048 | (-0.116;0.020) | 0.180 |
| Ambient PM_10_  (1-day mean) | -0.006 | (-0.014;-0.002) | 0.175 |  | -0.007 | (-0.018;0.004) | 0.211 |  | -0.002 | (-0.014;0.011) | 0.798 |
| Ambient PM_10_  (2-day mean) | -0.010 | (-0.020;-0.001) | **0.055** |  | -0.014 | (-0.027;-0.001) | **0.043** |  | -0.004 | (-0.020;0.012) | 0.604 |
| Ambient PM_10_  (5-day mean) | -0.021 | (-0.034;-0.008) | **0.003** |  | -0.020 | (-0.037;-0.003) | **0.028** |  | -0.020 | (-0.040;0.001) | **0.068** |
| Ambient PM_10_  (8-day mean) | -0.028 | (-0.045;-0.011) | **0.002** |  | -0.035 | (-0.060;-0.010) | **0.009** |  | -0.020 | (-0.045;0.006) | 0.137 |
| **Mitochondrial DNA Copy Number (%) in females** | | | | | | | | | | | |
| Personal PM_2.5_ (work hours) | 0.001 | (-0.016;0.018) | 0.879 |  | -0.004 | (-0.023;0.015) | 0.718 |  | 0.014 | (-0.018;0.045) | 0.406 |
| Personal EC (work hours) | -0.027 | (-0.146;0.091) | 0.655 |  | -0.049 | (-0.215;0.117) | 0.569 |  | 0.018 | (-0.166;0.202) | 0.851 |
| Ambient PM_10_  (1-day mean) | -0.005 | (-0.017;0.007) | 0.453 |  | -0.004 | (-0.021;0.012) | 0.609 |  | -0.006 | (-0.023;0.011) | 0.511 |
| Ambient PM_10_  (2-day mean) | -0.002 | (-0.016;0.012) | 0.767 |  | 0.001 | (-0.018;0.020) | 0.943 |  | -0.006 | (-0.025;0.014) | 0.579 |
| Ambient PM_10_  (5-day mean) | -0.001 | (-0.026;0.024) | 0.929 |  | 0.012 | (-0.024;0.047) | 0.524 |  | -0.011 | (-0.046;0.024) | 0.559 |
| Ambient PM_10_  (8-day mean) | 0.011 | (-0.034;0.056) | 0.645 |  | 0.023 | (-0.041;0.086) | 0.493 |  | -0.003 | (-0.068;0.062) | 0.931 |

**Table S3.** Pearson's Correlation Coefficient between MtDNAcn and risk factors

| **Risk factors** | **R^2^** | **p-value** |
| --- | --- | --- |
| Age | 0.081 | 0.212 |
| Pack-years of smoking | -0.134 | **0.038** |
| BMI | -0.127 | **0.049** |
| Systolic blood pressure | -0.203 | **0.002** |
| Diastolic blood pressure | -0.221 | **<0.001** |

**Table S4.** Pearson’s correlation coefficient among mtDNAcn, age, smoking, BMI and blood pressure

|  | **mtDNAcn** | **Age** | **Pack-years of smoking** | **BMI** | **Systolic blood pressure** | **Diastolic blood pressure** |
| --- | --- | --- | --- | --- | --- | --- |
| **mtDNAcn** | 1.000 |  |  |  |  |  |
|  |  |  |  |  |  |  |
| **Age** | 0.081 | 1.000 |  |  |  |  |
|  | *P=0.212* |  |  |  |  |  |
| **Pack-years of smoking** | -0.134 | 0.247 | 1.000 |  |  |  |
|  | *P=0.038* | *P=0.000* |  |  |  |  |
| **BMI** | -0.127 | 0.271 | 0.161 | 1.000 |  |  |
|  | *P=0.049* | *P<0.0001* | *P=0.012* |  |  |  |
| **SBP** | -0.203 | -0.041 | 0.205 | 0.428 | 1.000 |  |
|  | *P=0.002* | *P=0.523* | *P=0.001* | *P<0.0001* |  |  |
| **DBP** | -0.221 | 0.110 | 0.203 | 0.492 | 0.752 | 1.000 |
|  | *P=0.001* | *P=0.090* | *P=0.002* | *P<0.0001* | *P<0.0001* |  |

**Table S5.** Correlation between MtDNAcn and risk factors in bivariate regressions model^a^

| **Effect** | **Estimate** | **Standard Error** | **DF** | **t Value** | **Pr > |t|** | **Correlation Coefficient** |
| --- | --- | --- | --- | --- | --- | --- |
| **Pack years of smoking** | -0.004 | 0.003 | 117 | -1.37 | 0.174 | -0.112 |
| **BMI** | -0.009 | 0.006 | 117 | -1.50 | 0.137 | -0.120 |
| **SBP** | -0.003 | 0.001 | 118 | -2.43 | 0.016 | -0.168 |
| **DBP** | -0.005 | 0.002 | 118 | -2.78 | 0.006 | -0.185 |
| **Age** | 0.003 | 0.003 | 117 | 1.17 | 0.246 | 0.0942 |

^a^ Adjusted for occupation (office workers vs. truck drivers)
